# Relationship between genetically determined telomere length and childhood glioma risk

**DOI:** 10.1186/s40478-025-02199-2

**Published:** 2026-02-10

**Authors:** Naying Zhou, Shaobo Li, Jon Foss-Skiftesvik, Anna M. Dahlin, Jonas Bybjerg-Grauholm, Beatrice Melin, Adam J. de Smith, Kyle M. Walsh, Joseph L. Wiemels

**Affiliations:** 1 Department of Population and Public Health Science, Center for Genetic Epidemiology, Keck School of Medicine, University of Southern California, Los Angeles, CA USA; 2 Department of Neurosurgery, Rigshospitalet Copenhagen University Hospital, Copenhagen, Denmark; 3 Department of Diagnostics and Intervention, Oncology, Umeå University, Umeå, Sweden; 4 Department of Congenital Disorders, Statens Serum Institute, Copenhagen, Denmark; 5 Department of Neurosurgery and Duke Cancer Institute, Duke University School of Medicine, DUMC, Durham, NC USA

**Keywords:** Childhood cancer, Pediatric glioma, Mendelian randomization, Polygenic risk score, Leukocyte telomere length, Age–gene interaction

## Abstract

While longer genetically predicted leukocyte telomere length (LTL) has been linked to increased glioma risk in adults, this association has not been investigated in pediatric populations. In this study, we applied Mendelian randomization (MR) and polygenic risk score (PRS) analyses to investigate the relationship between LTL and pediatric glioma risk, using 4,069 cases and 8,778 controls from the largest available childhood glioma meta-GWAS. Results suggested longer genetically predicted LTL was significantly associated with increased childhood glioma risk, with OR per 1 standard deviation increase in LTL of 2.12 (95% CI 1.32–3.39, *P* = .002) in the multi-ancestry group and 2.16 (95% CI 1.16–4.03; *P* = .015) in the European group. Key SNPs contributing to risk of childhood glioma included rs59294613 (*POT1*), rs8105767 (*ZNF208*), and rs7705526 (*TERT*), which differed from the top variants previously identified in adult glioma using the same genetic instruments. Age-stratified MR revealed a stronger association in children diagnosed after the age of 6 (OR = 1.89; *P* < .001) vs. ≤ 6 years (OR = 1.04; *P* = .732; *P*_*het*_ = .006). PRS analysis further supported this age-stratified findings by demonstrating a positive association between LTL PRS and age at glioma diagnosis, particularly within age 0–10. This trend suggests a progressively greater influence of LTL on glioma risk in older children compared to younger children. In conclusion, this study provides the first genetic evidence linking longer genetically predicted LTL to increased pediatric glioma risk. While overall consistent with adult findings, distinct single-SNP associations, and age-dependent effects highlight unique biological mechanisms in children with glioma, warranting further direct investigation into telomere dynamics in early-life gliomagenesis.

**Supplementary Information:**

The online version contains supplementary material available at 10.1186/s40478-025-02199-2.

## Introduction

Brain and other central nervous system (CNS) tumors embody the second most common type of cancer in children and adolescents, accounting for 25% of all cancer cases diagnosed before 20 years of age [1]. 

Gliomas are the most common CNS tumors, accounting for approximately 43% of cases in children and adolescents aged 0–19 years and 23% across all age groups [2]. Although gliomas occur in both children and adults, their subtype distributions differ markedly. In children, low-grade gliomas (LGGs) constitute nearly two-thirds of all glioma cases, whereas in adults, high-grade gliomas (HGGs) are far more common (85% of all gliomas) [3, 4]. In pediatrics, HGGs carry high mortality and are a major driver of deaths from pediatric brain tumors, which collectively are the leading cause of cancer-related death in children [5]. By contrast, pediatric LGGs may often be managed as persistent conditions without immediate surgery; however, with prolonged survival, patients often experience substantial morbidity related to both the disease and its treatment [6–8].

Given the substantial impact of gliomas across lifespan, a deeper understanding of the factors contributing to glioma development is critical. To date, only a few risk factors for glioma show consistent evidence [9]. Therapeutic cranial radiation is the most consistently associated environmental risk factor [10–12]. Genetically, rare Mendelian disorders, such as Li-Fraumeni syndrome, neurofibromatosis type 1, and tuberous sclerosis complex, are linked to increased susceptibility [13–15]. Additionally, population-based sampling has facilitated genome-wide association studies (GWAS), which have identified single nucleotide polymorphisms (SNPs) associated with glioma risk [16, 17]. The largest adult meta-GWAS combined eight international studies (12,496 cases; 18,190 controls; mean diagnosis age around 47 years) and reported 38 genome-wide significant risk loci [16]. Recent work has extended these findings using region-based analyses [18], integration with multi-omics data [19], and GWAS focused on molecularly defined diffuse glioma subtypes [20]. In contrast, childhood gliomas are rarer. The largest pediatric meta-GWAS to date combined three cohorts from the United States, Sweden, and Denmark (4,069 cases; 8,778 controls; ages 0–19) and identified a significant association at CDKN2B-AS1 (9p21.3) for astrocytoma [17]. This locus is concordant with adult glioma findings, whereas additional genome-wide significant loci identified in adults have not, to our knowledge, shown comparable signals in pediatric analyses, suggesting age-dependent genetic architecture. The growing availability of GWAS data across a wide range of traits has further enabled the application of Mendelian randomization (MR) analysis, which is a powerful approach for assessing causal relationships between risk factors and glioma risk using SNPs as genetic instruments [21].

Telomeres are repetitive DNA sequences located at the ends of chromosomes, essential for maintaining genomic stability during cell division. In 1998, Bodnar et al. first demonstrated telomerase activation as a key mechanism for bypassing cellular senescence [22]. Subsequent studies linked somatic mutations in the *TERT* gene to both pediatric and adult glioma, and telomerase-related SNPs have been associated not only with increased glioma risk but also with older age at diagnosis in adult patients [23–27]. Furthermore, leukocyte telomere length (LTL) was first reported to be associated with glioma risk in a case–control study by Wang et al. [28]. Later MR analyses have strengthened the evidence for a causal relationship between genetically predicted longer LTL and increased glioma risk in adults [29–31]. As large-scale GWAS data have become more available, multiple MR studies have produced consistent findings [32–34]. However, these MR studies have primarily focused on adult populations, and the association between LTL and pediatric glioma remains unclear.

Given the distinct molecular landscape and epidemiology of gliomas [8, 35–37], as well as the differences in telomere dynamics between children and adults [32, 38, 39], it is important to investigate whether the genetic factors identified in adult glioma also contribute to disease risk in pediatric populations. In this study, we leveraged large-scale GWAS data and applied MR and polygenic risk scores (PRS) analysis to examine the relationship between genetically predicted LTL and the risk of childhood glioma.

## Methods

### Genetic instruments for leukocyte telomere length

Genetic instruments for LTL were selected from two published GWAS [40, 41]. Fifteen of the 20 SNPs reported by Li et al. were used as the LTL genetic instrument for adult glioma MR analyses. The same SNPs were evaluated in our childhood glioma MR analyses using meta-GWAS results, enabling direct comparison with prior adult studies [40]. An additional genetic instrument from Burren et al., based on a joint telomere length metric combining qPCR and whole-genome sequencing measurements, was incorporated [41]. This genetic instrument explained approximately 10% of LTL heritability and was employed to evaluate associations in age-stratified analyses to increase statistical power in strata with smaller sample sizes. Variants reaching genome-wide significance (*P* < 5 × 10^−8^) and with minor allele frequency > 0.01 were retained, followed by linkage disequilibrium pruning (r^2^ < 0.05), resulting in 118 of the 192 SNPs retained for this study.

### Childhood glioma datasets

Childhood glioma GWAS summary statistics were obtained from a meta-analysis of three cohorts (the U.S., Sweden, and Denmark), comprising 4,069 cases and 8,778 controls aged 0–19 years and representing diverse ancestral backgrounds [17]. The U.S. cohort included 2,310 pediatric glioma cases and 3,185 controls. With individual-level genotyping data and detailed age-at-diagnosis records, we performed PRS and age-stratified MR analyses in this cohort. Demographic and phenotypic characteristics of the U.S. cohort by ancestry group were described in Table S1. Diagnostic criteria, quality control, and imputation of this cohort followed the original study [17].

### Mendelian randomization analysis

The primary MR analysis used a random-effects inverse-variance weighted (IVW) model, supplemented by weighted median, weighted mode, and single-SNP (Wald ratio) analyses [42, 43]. For single-SNP MR analyses, multiple testing was addressed using the Benjamini-Hochberg false discovery procedure. For the assumptions of MR, we assessed heterogeneity (Cochran’s Q), horizontal pleiotropy (MR-Egger intercept, MRClust), and conducted leave-one-out analyses [44–47]. Analyses were stratified by ancestry (European vs. full cohort), glioma subtype, and age at diagnosis. All MR analyses were conducted in R (v4.2.2) using the TwoSampleMR package [46].

### Polygenic risk score analysis

Using SNPs from Burren et al., individual-level LTL PRS were calculated with PRSice-2 (v2.3.5) following Choi et al.’s guidelines [41, 48, 49], and analyzed in the U.S. cohort. Regression analyses assessed associations between PRS and glioma risk, and between PRS and diagnosis-age. Principal component analysis was performed on individual-level genotyping data to capture genetic population structure, and the first 10 principal components (PCs) were included as covariates in regression models. This number was selected to control for major ancestry-related variation while minimizing overfitting, and is consistent with previous childhood glioma GWAS meta-analyses. All models were adjusted for sex and the first 10 PCs, with diagnostic checks performed to validate assumptions. A one-way ANOVA (F-test) was used to compare mean LTL PRS among ancestry groups. Generalized Additive Model (GAM) was used to examine non-linear associations between LTL PRS and age at diagnosis [50]. Analyses were conducted in R (v4.2.2).

**Fig. 1:**
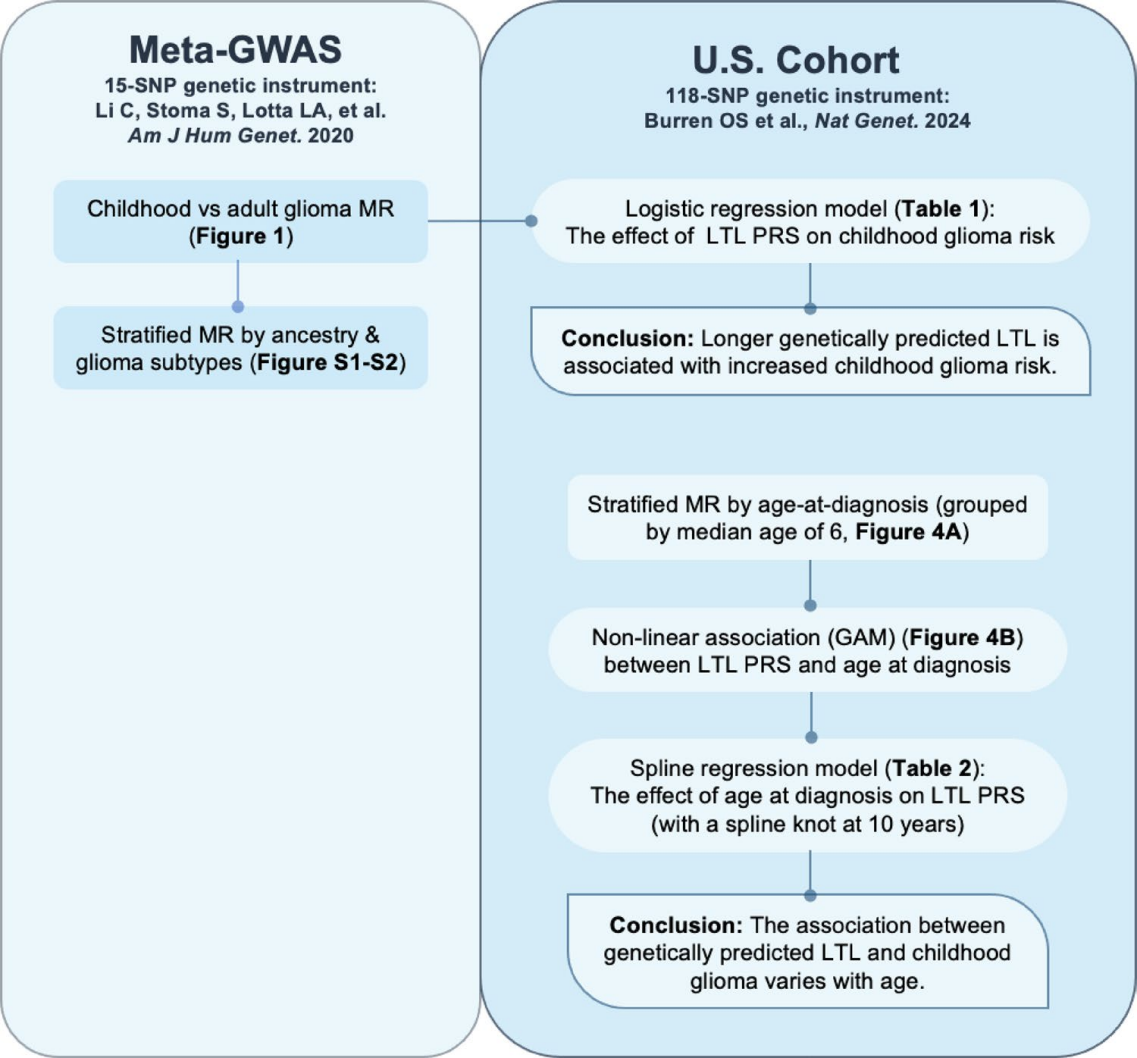
Overview of study design. The left panel shows analyses using childhood glioma meta-GWAS data with a genetic instrument comprising 15 SNPs [40]. The right panel shows analyses in the U.S. childhood glioma cohort using a genetic instrument with 118 SNPs [41]. Within each panel, square boxes denote MR analyses, rounded boxes denote PRS analyses, and boxes outlined with solid lines indicate key conclusions. GWAS, Genome-wide association study; SNPs, Single nucleotide polymorphisms; MR, Mendelian randomization; LTL, Leukocyte telomere length; PRS, Polygenic risk score; GAM, Generalized additive model

The dataset, genetic instruments, and corresponding analyses are summarized in Fig. 1.

## Results

**Fig. 2:**
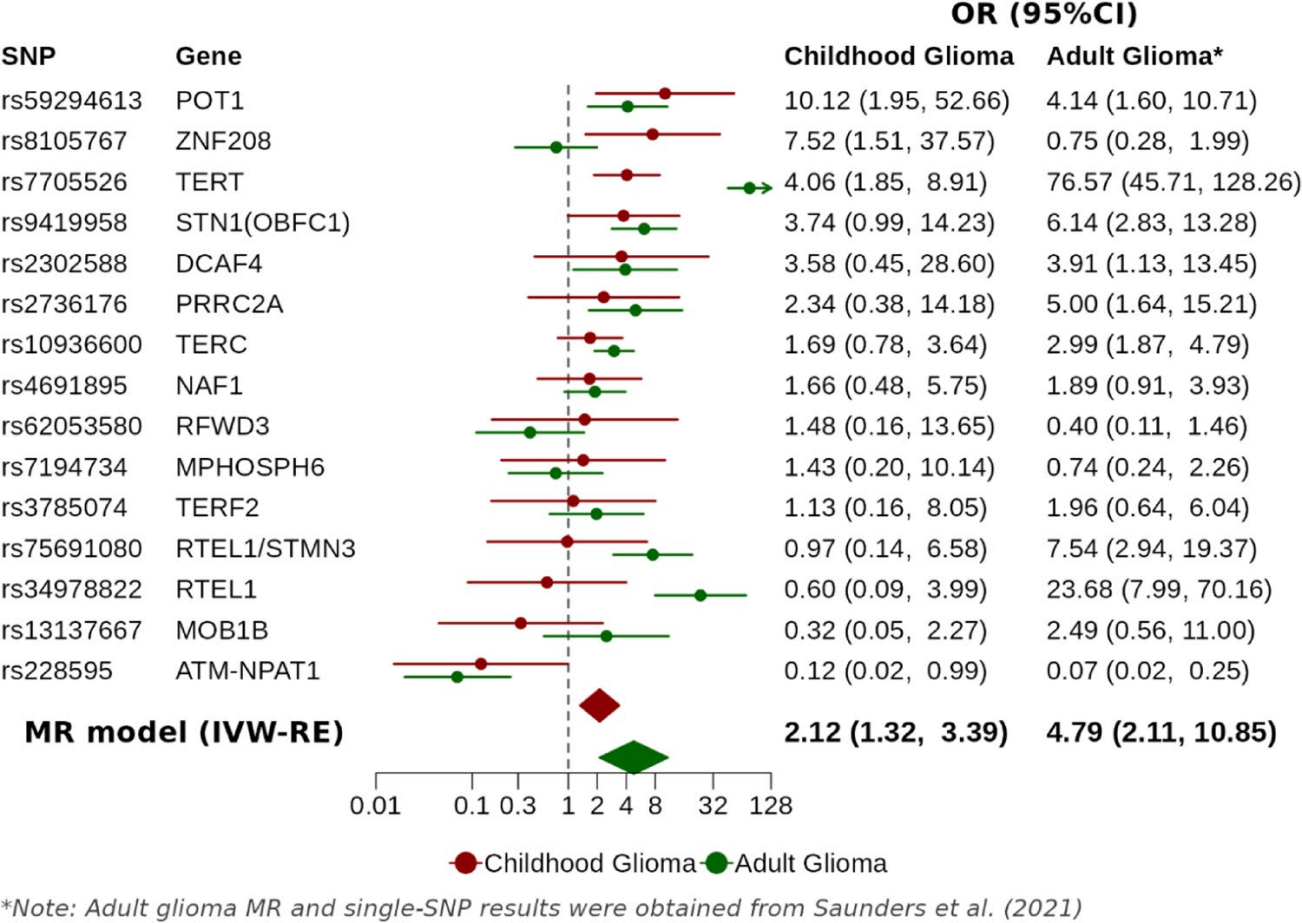
Forest plot comparing MR and single-SNP analysis results for the association between genetically predicted LTL and the risk of childhood and adult glioma. Adult glioma MR and single-SNP results (*) were obtained from Saunders et al. [32]. Among the 15 SNPs associated with LTL, rs59294613 (*POT1*) showed the strongest association with pediatric gliomas, with a greater impact on overall MR results compared to its impact in adult gliomas. In contrast, the key associations identified in adult glioma were rs7705526 (*TERT*), rs34978822 (*RTEL1*), and rs75691080 (*RTEL1/STMN3*). Notably, rs7705526 (*TERT*) was the only SNP with a high effect size in both childhood and adult glioma. *RTEL1* was the second most associated LTL SNP in adult gliomas but showed a null effect in pediatric gliomas. Similarly, *ZNF208*, which had the second highest OR in childhood glioma was not a significant glioma risk variant in adults. Results were reported as ORs with 95% CIs per genetically predicted one SD increase in directly measured telomere length [40]. The dashed vertical line at OR = 1. Red and green denote results from childhood glioma study and adult glioma study, respectively. IVW-RE, Inverse-variance weighted random-effects; MR, Mendelian randomization; FDR, False discovery rate; OR, Odds ratio; CIs, Confidence intervals; SD, Standard deviation

We observed a significant relationship between increased genetically predicted LTL and elevated risk of childhood glioma using IVW-RE model, with ORs per SD increase in predicted LTL of 2.12 (95% CI 1.32–3.39, *P* = .002) (Fig. 2). Comparison across different MR models (IVW-RE, weighted median, weighted mode) in both multi-ancestry (all) and European ancestry analyses revealed consistent results (Fig. S1). Subtype-specific childhood glioma MR results suggested that the subgroup MR effect sizes are consistently above one across all groups, except for oligodendroglioma (Fig. S2). Notably, low-grade glioma exhibited larger effect sizes compared to high-grade glioma in both the multi-ancestry and European groups. However, the interpretation of results for subgroups that include high-grade glioma, glioblastoma, and oligodendroglioma was limited due to more modest sample sizes in these groups. The leave-one-out analysis indicated that no single SNP was responsible for the observed relationships (Fig. S3). Significant heterogeneity was detected in the fixed-effect IVW model (Table S2, *P* = .05). As a result, the random-effects IVW model was used for the analysis.

The association between childhood glioma risk and each of the 15 LTL SNPs included in the genetic instrument of MR analysis were tested, with three SNPs with significant LTL-associated childhood glioma risk (i.e., Benjamini-Hochberg FDR q <  = .1) identified: rs59294613 at 7q31.33 (*POT1*), rs8105767 at 19p12 (*ZNF208*), and rs7705526 at 5p15.33 (*TERT*) (Fig. 2).The results exhibited different coefficient rankings compared to adult glioma study [32]. Additionally, none of the 15 SNPs reached the genome-wide significance threshold (*P* < 5 × 10^−8^) in childhood glioma GWAS. The lack of strong individual SNP associations with childhood glioma, combined with a non-significant MR-Egger intercept test and the identification of a single cluster by MR-Clust, suggests a minimal risk of horizontal pleiotropy (Fig. S4, Table S3).

**Fig. 3:**
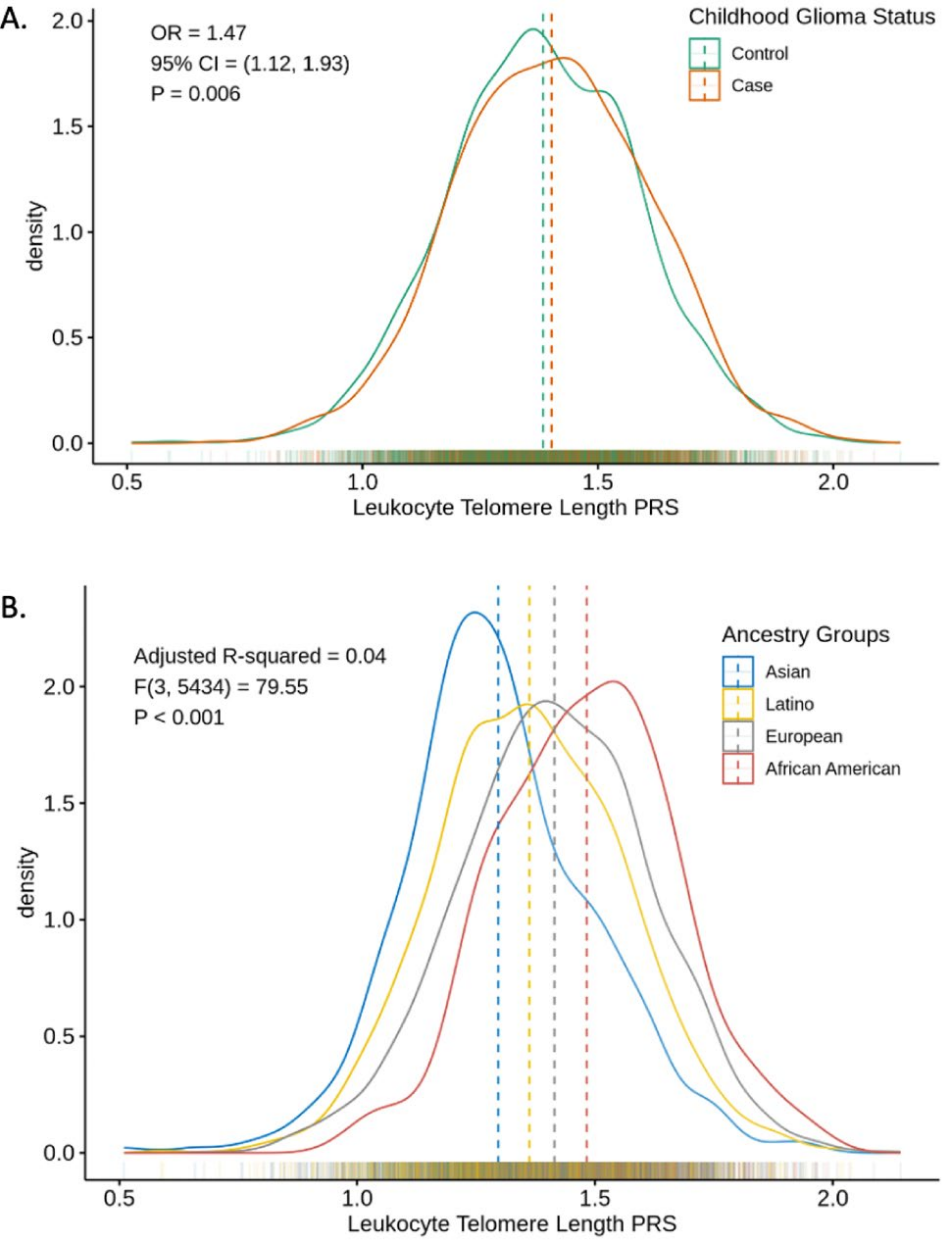
Density plots of LTL polygenic risk scores. **A** Comparison between childhood glioma cases and controls. OR was estimated using logistic regression adjusted for sex and the first 10 genetic PCs from the childhood glioma genotyping data. **B** Comparison across ancestry groups. A one-way ANOVA (F-test) was used to compare mean LTL PRS among ancestry groups. Dashed lines indicate the mean LTL PRS values within each group. OR, Odds ratio; PCs, Principal components; ANOVA, Analysis of variance; LTL PRS, Leukocyte telomere length polygenic risk score

PRS analysis was conducted in the U.S. cohort, incorporating 118-SNP genetic instrument identified by Burren et al. [41] (Fig. 3A). Logistic regression analysis of childhood glioma status was performed with LTL PRS as the independent variable, adjusting for sex and first 10 genetic PCs (Table 1). LTL PRS distributions were significantly different among ancestry groups regardless of childhood glioma status (*P* < .001, Fig. 3B). Since the LTL genetic instrument was developed in European population, cross-population differences in allele frequency and linkage disequilibrium may reduce instrument performance in other ancestry groups. Consequently, logistic regression analyses were conducted across all data and within different ancestry groups to assess the association between LTL PRS and childhood glioma status (Table 1, Fig. S5). In the overall cohort, each unit increase in LTL PRS was associated with an increased childhood glioma risk (OR = 1.47, 95% CI 1.12–1.93, *P* = .006). In the European group, the risk increase was more pronounced, with each unit increase in LTL PRS linked to 1.72 times the odds of developing childhood glioma (95% CI 1.17–2.53, *P* = .006). The Asian group showed the highest OR of 1.95 (95% CI 0.61–6.35, *P* = .265). However, no significant association was observed between LTL PRS and childhood glioma status in Latino, Asian, or African American groups. Combining with Fig. 3B, these null findings likely reflect limited sample sizes and reduced generalizability of the LTL genetic instrument to other ancestry groups. Similar analyses were conducted for each glioma subtype, with a significant result observed only in the astrocytoma group. A one-unit increase in LTL PRS was associated with a 1.4-fold increase in the odds of developing astrocytoma (95% CI 1.03–1.89, *P* = .03, Table S4).

**Table 1:** Logistic regression results for the association between LTL PRS and childhood glioma status, stratified by ancestry group

Ancestry	LTL PRS			
	N (case/control)	OR (95% CI)	AIC	*P*
Multi-ancestry	2310/3185	1.47 (1.12, 1.93)	7464.50	0.006
European	1142/1559	1.72 (1.17, 2.53)	3650.21	0.006
Latino	823/1177	1.22 (0.78, 1.93)	2717.68	0.380
Asian	168/222	1.95 (0.61, 6.35)	534.88	0.265
African American	151/196	1.07 (0.33, 3.48)	495.07	0.907

Odds ratios (ORs) per one-unit increase in LTL PRS were estimated using logistic regression models adjusted for sex and the first 10 genetic PCs. ORs are presented with 95% confidence intervals (CIs) and corresponding *P* values

LTL PRS, Leukocyte telomere length polygenic risk score; OR, Odds ratio; CIs, Confidence intervals; AIC, Akaike Information Criterion; PCs, Principal components

**Fig. 4:**
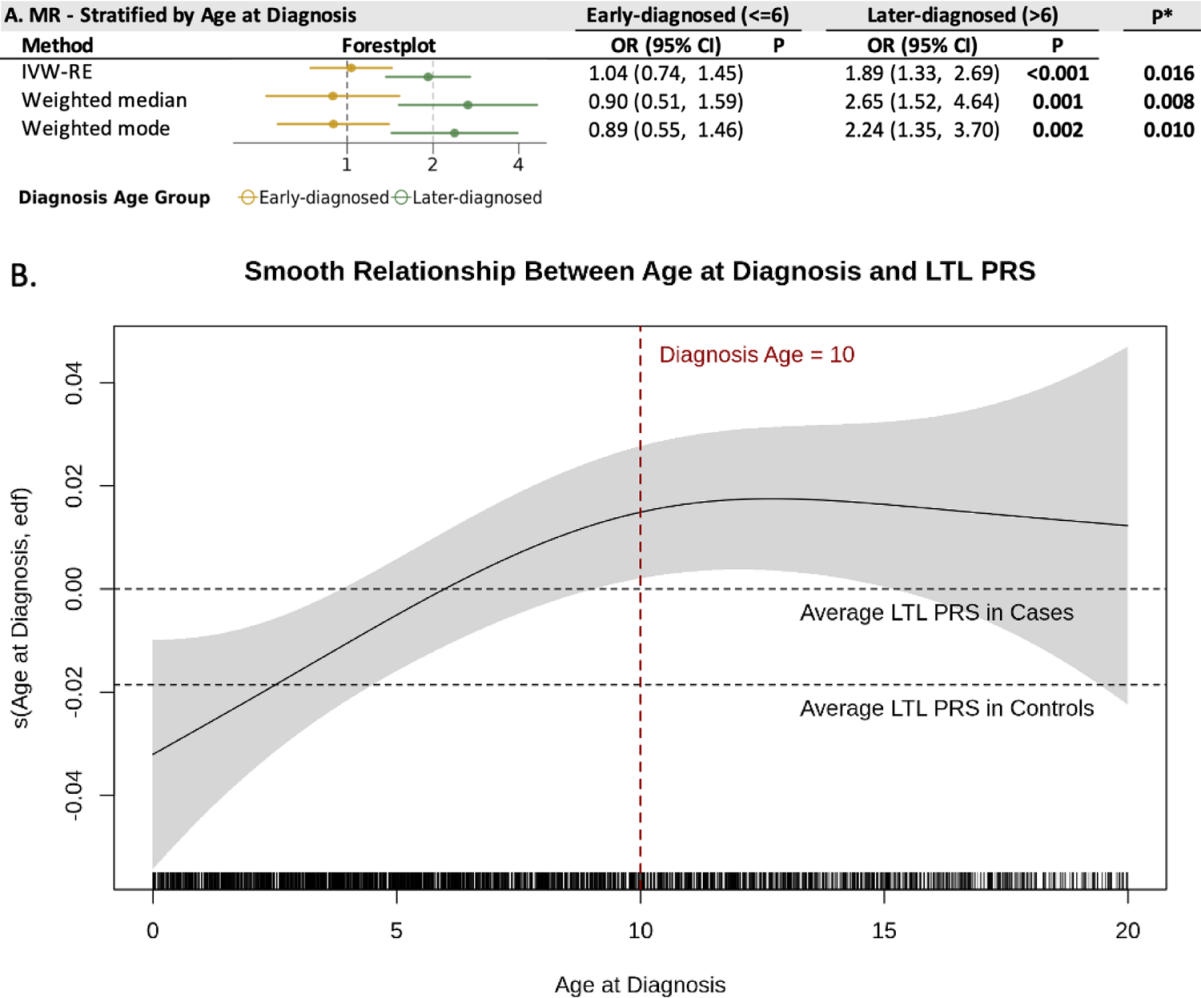
Stratified MR analysis and LTL PRS analysis by age at diagnosis. **A** Forest plot of MR estimates for each age-at-diagnosis group. Results were obtained using inverse-variance weighted (random-effects, IVW-RE), weighted median, and weighted mode models. Results were reported as odds ratios (ORs) with 95% confidence intervals (CIs) per genetically predicted one standard deviation (SD) increase in telomere length, measured via a combined metric [41]. P* represents heterogeneity tests between groups. **B** Generalized additive model (GAM) illustrating the smoothed association between LTL PRS and age at diagnosis, adjusted for sex and the first 10 principal components. The red vertical line marks age 10 years; black horizontal lines indicate the mean LTL-PRS in childhood glioma cases and controls. The y-axis shows the difference between the mean LTL-PRS among cases at a given diagnosis age and the overall mean LTL-PRS among cases. At younger diagnosis ages, case means are closer to control means. The plot indicates that the association between LTL and childhood glioma strengthens from ages 0–10 and then stabilizes, consistent with age-dependent variation in the LTL-childhood glioma association. The slight downward trend beyond age 15 likely reflects imprecision due to smaller sample sizes at older ages. Dashed lines indicate the mean PRS values within each group. MR, Mendelian randomization; GAM, Generalized additive model; LTL PRS, Leukocyte telomere length polygenic risk score

 We further examined the association between genetically predicted LTL and childhood glioma risk across different age at diagnosis groups (Fig. 4). In the U.S. cohort, glioma cases were stratified into early-diagnosed (≤ 6 years) and later-diagnosed (> 6 years) groups based on the median age to ensure balanced sample sizes (Table S1). Analytical power was further improved by using a genetic instrument comprising 118 LTL-associated SNPs identified by Burren et al. [41]. As shown in Fig. 4A, OR per SD is 1.89 (95% CI 1.33–2.69; *P* < .001) and 1.04 (95% CI 0.74–1.45; *P* = .73) in later-diagnosed and early-diagnosed groups, respectively (P_het_ = .006). MR heterogeneity tests, pleiotropy tests, and leave-one-out analysis all shows insignificant results (Tables S2 and S3, Fig. S6).

To further explore potential trends and non-linear relationships between LTL PRS and diagnosis age, we applied a generalized additive model (GAM) in childhood glioma cases. As shown in Fig. 4B, the fitted smooth function of LTL PRS increases steadily for diagnosis ages 0–10, suggesting a positive association between LTL PRS and diagnosis age. Beyond age 10, the curve plateaus, indicating diminishing differences in average PRS among children diagnosed later. The confidence interval expands for diagnosis ages < 3 and > 15, reflecting larger variations due to the limited number of glioma cases in these age ranges. This observed non-linear relationship suggests age-dependent variation in the association between LTL and childhood glioma.

Guided by the GAM and to further quantify the pattern observed in Fig. 4B, a spline regression model was fitted among childhood glioma cases, modeling LTL-PRS as the outcome and age at diagnosis (in months) as the predictor, with a knot at 10 years (Table 2). Among cases diagnosed before age 10, age at diagnosis was positively associated with LTL-PRS (*P* < .001), indicating that the LTL-childhood glioma association strengthens with increasing age. Among cases diagnosed after 10 years, the association was null (*P* = .697), suggesting the relationship between LTL and risk of childhood glioma becomes stable after 10. This result is consistent with the stratified diagnosis age group MR analyses (Fig. 4A), although different age cutoff points were applied. In ancestry-stratified analyses, similar patterns were observed in the European and Latino subgroups but not in the Asian or African American subgroups, likely reflecting limited statistical power in the smaller ancestry strata. (Table 2).

**Table 2:** Spline regression model results of childhood glioma diagnosis age on LTL PRS, stratified by ancestry group

Ancestry	Diagnosis age ≤ 10			Diagnosis age > 10		
	Number of cases	β (95% CI)	*P*	Number of cases	β (95% CI)	*P*
Multi-ancestry	1588	0.100 (0.052, 0.149)	< .001	709	0.007 (− 0.029, 0.043)	.697
European	765	0.101 (0.029, 0.173)	.006	369	0.000 (− 0.048, 0.048)	.995
Latino	589	0.118 (0.037, 0.198)	.004	231	0.001 (− 0.065, 0.067)	.972
Asian	112	0.102 (− 0.066, 0.271)	.233	55	0.126 (− 0.018, 0.270)	.087
African American	104	0.007 (− 0.197, 0.211)	.945	47	0.008 (− 0.135, 0.152)	.908

Age at diagnosis was modeled in months with a spline knot at 10 years, and models were adjusted for sex and the first 10 genetic principal components. Similar patterns were observed in the European and Latino subgroups but not in the Asian or African American subgroups, likely reflecting limited statistical power in the smaller ancestry strata. Coefficients (β) are shown with 95% CIs and *P* values. Results indicate a positive association before age 10 years and no association thereafter

LTL PRS, Leukocyte telomere length polygenic risk score; PCs, Principal components; CIs, Confidence intervals

## Discussion and conclusions

This study provides the first genetic evidence linking leukocyte telomere length to glioma risk in a pediatric population. We found that longer genetically predicted LTL is associated with an increased risk of childhood glioma, consistent with findings in adult glioma [29–32]. Stratified analyses further revealed that this association is primarily driven by cases diagnosed later in childhood, indicating age-dependent variation in the LTL–childhood glioma relationship. These findings not only refine our understanding of leukocyte telomere length in pediatric glioma etiology but also introduce new insights into its non-linear association with diagnosis age, which may have implications for future risk stratification, early detection, and treatment guidance.

Telomere length, determined by the balance between elongation and maintenance, plays a critical role in glioma development and progression [23–27]. Recent GWAS have identified key variants associated with LTL maintenance, revealing the genetic complexity underlying LTL regulation [40, 41]. These variants from GWAS were used as genetic instruments in MR analyses to provide a proxy assessment of the relationship between longer LTL and increased glioma risk. For MR estimates to be unbiased, three core assumptions must be met: the genetic instrument must be strongly associated with the exposure, independent of confounders, and not directly affect the outcome outside of the phenotype estimated by the instrument [21]. To ensure validity of MR assumptions in our childhood glioma cohort, we confirmed that none of the genetic instruments were significantly associated with childhood glioma in our GWAS, and the MR-Egger intercept showed no evidence of horizontal pleiotropy. MR-Clust identified a single non-null cluster, indicating a shared causal pathway. Together, these results support the validity of the instruments and suggest they are not confounded by alternative biological pathways. Overall, our MR findings are considered valid and align with prior evidence from adult glioma studies, suggesting that longer LTL may be a shared genetic risk factor of glioma across age groups.

To further explore the observed association and assess heterogeneity, we performed single-SNP analyses. Comparison with adult glioma single-SNP results revealed distinct differences between the two populations [32], and the key SNPs driving these differences are discussed in detail below.

rs59294613 (*POT1*) exhibited the strongest association with childhood glioma in single-SNP analyses, suggesting a greater influence on the overall MR estimates in children than in adults, indicating a more prominent role in early-onset glioma [32], Similarly, the *ZNF208* variant rs8105767 was the second most significant SNP in the single-SNP analysis for childhood glioma but is not associated with adult glioma risk itself. *ZNF208* rs8105767 is reported to be associated with a favorable prognosis in patients with low-grade glioma [51], which aligns with the higher proportion of WHO grade I pilocytic astrocytoma with a better prognosis in pediatric glioma [52].

*TERT* and *RTEL1* have well-established genetic associations with adult glioma, playing a critical role in glioma progression through the telomere maintenance mechanism [25, 26, 53]. An MR study by Saunders et al. supports this association by identifying *TERT* rs7705526, *RTEL1* rs34978822 and *RTEL1/STMN3* rs75691080 as the top three LTL-related variants with the largest effect sizes in single-SNP tests linked to increased adult glioma risk [32]. However, we observed distinct results for these loci in our study of childhood glioma. While *TERT* rs7705526 remains associated with an increased risk of childhood glioma, its effect size is significantly smaller than that for adult glioma (Wald test, *P* < .001). In addition, SNPs in *RTEL1* and *RTEL1/STMN3* show no association with childhood glioma. Koelsche et al.'s study further supports our findings, demonstrating that *TERT* promoter mutations are uncommon in pediatric nervous system tumors relative to adult cases and, when detected, are more likely to occur in older children [26].

In conclusion, the comparison of single-SNP effects between pediatric and adult population highlights the potential age-related genetic heterogeneity in glioma susceptibility associated with telomere length. This may result from distinct mechanisms by which longer telomeres influence glioma risk in children and adults [27]. Additionally, GWAS estimates based solely on adults may reflect age-conditional rather than age-independent genetic effects. Consequently, SNP effects derived from adult cohorts may not generalize across the lifespan, as gene-age interactions could shape telomere length associations at younger ages in ways unobserved in adult-only samples [40]. To further investigate age-LTL interactions, stratified MR analyses were conducted in children, revealing a significant association in later-diagnosed children (> 6 years) but not in early-diagnosed cases (≤ 6 years). These findings are consistent with a prior study by Walsh et al., in which LTL PRS and diagnosis age were modeled in childhood and adolescent ependymoma patients derived from the same California population as the U.S. cohort in the current study, and provide additional evidence for an age-related interaction effect [54].

A deeper analysis of childhood glioma cases revealed a changing pattern in average LTL PRS with diagnosis age, with LTL PRS increasing from ages 0 to 10 before plateauing. This pattern may reflect the limitations of adult-based genetic instruments in capturing early-life telomere dynamics, given previous evidence of differed pattern of LTL shortening in early life [38, 55]. Cowell et al.’s longitudinal study reported rapid LTL shortening from birth to age 3, a slower decline between ages 3 and 5, and becoming stable by age 9 [39]. The varied LTL decreasing rates suggest potential differences in the genetic regulation of LTL between young children and adults.

A related consideration is the role of obesity in the LTL–childhood glioma relationship. Obesity has been linked to shorter LTL in both children and adults, and lifestyle or weight-loss interventions have been associated with increases in peripheral LTL, suggesting partial reversibility [56–59]. Some studies also suggest obesity may increase glioma risk, although findings are inconsistent [60, 61]. In our study, we leveraged genetically predicted LTL using instruments from adult LTL GWAS and pediatric genotypes obtained from neonatal dried blood spots, thereby minimizing confounding by obesity status at the time of glioma diagnosis.

While our findings provide valuable insights, there are a few caveats that should be noted. First, in analysis stratified by ancestry group, the association between predicted LTL and childhood glioma risk was only significant in the European ancestry group, although positive ORs were detected in all groups. The smaller sample size of the non-European ancestry groups, in particular for Asians and African Americans, would have reduced our statistical power to detect associations. It is also important to highlight, however, that the LTL related SNPs were derived from GWAS conducted in a predominantly European ancestry population, and therefore might not be ideally applicable in other ancestry groups. Subtype glioma MR analyses have encountered similar sample size issues, which might explain inconsistent results of previously reported critical role of telomere length in glioblastoma with our analyses (our GBM n = 84) [27].

Secondly, the methodological limitations of MR warrant careful consideration. A point of discussion is whether rs59294613 in the *POT1* gene violates MR assumptions, given previous studies establishing a strong link between *POT1* germline variants and familial glioma [62]. *POT1* (Protection of Telomeres 1) encodes a key component of the shelterin complex, which is critical for telomere capping and length regulation [63–65]. Variants in *POT1* that predispose to cancer are typically associated with longer, but structurally fragile, telomeres [66–68]. Although *POT1* has been implicated in familial glioma, we consider its effect on childhood glioma risk in this study to be mediated through telomere length regulation. Therefore, it is unlikely that this variant violates core MR assumptions such as a direct association with the outcome or influence through alternative, confounding pathways. In addition, our study primarily focuses on the combined marginal effects of common alleles, whereas rare pathogenic germline mutations in *POT1* are outside the scope of our investigation.

Finally, the genetic instrument based on LTL measured in adult populations may be subject to bias due to pre-existing and unaccounted age–gene interactions. Moreover, current GWAS analyses measuring adult LTL cannot fully capture the genetic factors influencing early-life LTL dynamics, highlighting the need for large-scale GWAS studies incorporating laboratory measurements of telomere length in pediatric populations.

In summary, our findings support a positive relationship between longer genetically predicted LTL and increased risk of childhood glioma. However, two key observations highlight the complexity of this association. First, differences in single-SNP effects between pediatric and adult glioma suggest potential age-related genetic heterogeneity of this relationship. Second, the stronger association observed in later-diagnosed children points to age-dependent variation in the LTL-childhood glioma relationship. These findings underscore the need for further telomere-focused research on glioma risk, particularly in early-life contexts.

## Supplementary Information

Below is the link to the electronic supplementary material.

Supplementary Material 1

## Data Availability

(1) Childhood glioma GWAS data were obtained from a recent published meta-analysis of three population-based cohorts from the United States, Sweden, and Denmark. The GWAS summary statistics used in our analysis are available on the Havard dataverse website (https://dataverse.harvard.edu/previewurl.xhtml?token=7045b3a7-f00d-4cc5-8507-c4e94574afa3). (2) Genetic instruments were derived from two published large-scale GWAS of leukocyte telomere length. (3) Our individual-level childhood glioma genotyping data is derived from the California Biobank and are not publicly available due to the below reasons: We respectfully are unable to share raw, individual genetic data freely with other investigators since the samples and the data are the property of the State of California. Should we be contacted by other investigators who would like to use the data, we will direct them to the California Department of Public Health Institutional Review Board to establish their own approved protocol to utilize the data, which can then be shared peer-to-peer. The State has provided guidance on data sharing noted in the statement below: “California has determined that researchers requesting the use of California Biobank biospecimens for their studies will need to seek an exemption from NIH or other granting or funder requirements regarding the uploading of study results into an external bank or repository (including into the NIH dbGaP or other bank or repository). This applies to any uploading of genomic data and/or sharing of these biospecimens or individual data derived from these biospecimens. Such activities have been determined to violate the statutory scheme at California Health and Safety Code Section 124980 (j), 124991 (b), (g), (h) and 103850 (a) and (d), which protect the confidential nature of biospecimens and individual data derived from biospecimens. Investigators may agree to share aggregate data on SNP frequency and their associated *P* values with other investigators and may upload such frequencies into repositories including the NIH dbGaP repository providing: (a) the denominator from which the data is derived includes no fewer than 20,000 individuals; (b) no cell count is for < 5 individuals; and (c) no correlations or linkage probabilities between SNPs are provided”. The collection of cancer incidence data used in this study was supported by the California Department of Public Health as part of the statewide cancer reporting program mandated by California Health and Safety Code Section 103885; the National Cancer Institute’s Surveillance, Epidemiology and End Results Program under contract HHSN261201000140C awarded to the Cancer Prevention Institute of California, contract HHSN261201000035C awarded to the University of Southern California, and contract HHSN261201000034C awarded to the Public Health Institute; and the Centers for Disease Control and Prevention’s National Program of Cancer Registries, under agreement U58DP003862-01 awarded to the California Department of Public Health. The biospecimens and/or data used in this study (for California data) were obtained from the California Biobank Program, (SIS request #311), Section 6555 (b), 17 CCR. The California Department of Public Health is not responsible for the results or conclusions drawn by the authors of this publication.

## Abbreviations

LTL: Leukocyte telomere length
MR: Mendelian randomization
PRS: Polygenic risk score
GWAS: Genome-wide association study
SNP: Single nucleotide polymorphism
LD: Linkage disequilibrium
MAF: Minor allele frequency
IVW: Inverse variance weighted
