# Supplementary material for: Relationship between genetically determined telomere length and childhood glioma risk
Source: Acta Neuropathol Commun. 2026 Feb 10;14:93. doi: 10.1186/s40478-025-02199-2 (PMC13085377; doi:10.1186/s40478-025-02199-2)
Supplement: Supplementary file 1 — Supplementary Material 1 [file 40478_2025_2199_MOESM1_ESM.docx]

Supplementary material:

Table of Contents

[Supplementary Figures: 2](#_Toc213933189)

[Figure S1. Forest plot of MR and single-SNP analysis of LTL effects on childhood glioma (all vs European ancestry) 2](#_Toc213933190)

[Figure S2. Forest plot of MR analysis of LTL effects on childhood glioma subtypes (all vs European ancestry) 3](#_Toc213933191)

[Figure S3. Sensitivity MR analyses supporting main MR findings in Figure 2 4](#_Toc213933192)

[Figure S4. MR-Clust results showing SNP clustering based on LTL and childhood glioma associations 5](#_Toc213933193)

[Figure S5 Density plots of LTL PRS by glioma status across four ancestry groups 6](#_Toc213933194)

[Figure S6. Sensitivity MR analyses supporting age-stratified MR findings in Figure 4A 7](#_Toc213933195)

[Supplementary tables: 8](#_Toc213933196)

[Table S1. Demographic and phenotypic characteristics of the US cohort by ancestry group. 8](#_Toc213933197)

[Table S2. IVW heterogeneity test results 8](#_Toc213933198)

[Table S3. Directional horizontal pleiotropy test 9](#_Toc213933199)

[Table S4. Logistic regression results for the association between LTL PRS and childhood glioma status, stratified by glioma subtypes. 10](#_Toc213933200)

# Supplementary Figures:

## Figure S1. Forest plot of MR and single-SNP analysis of LTL effects on childhood glioma (all vs European ancestry)


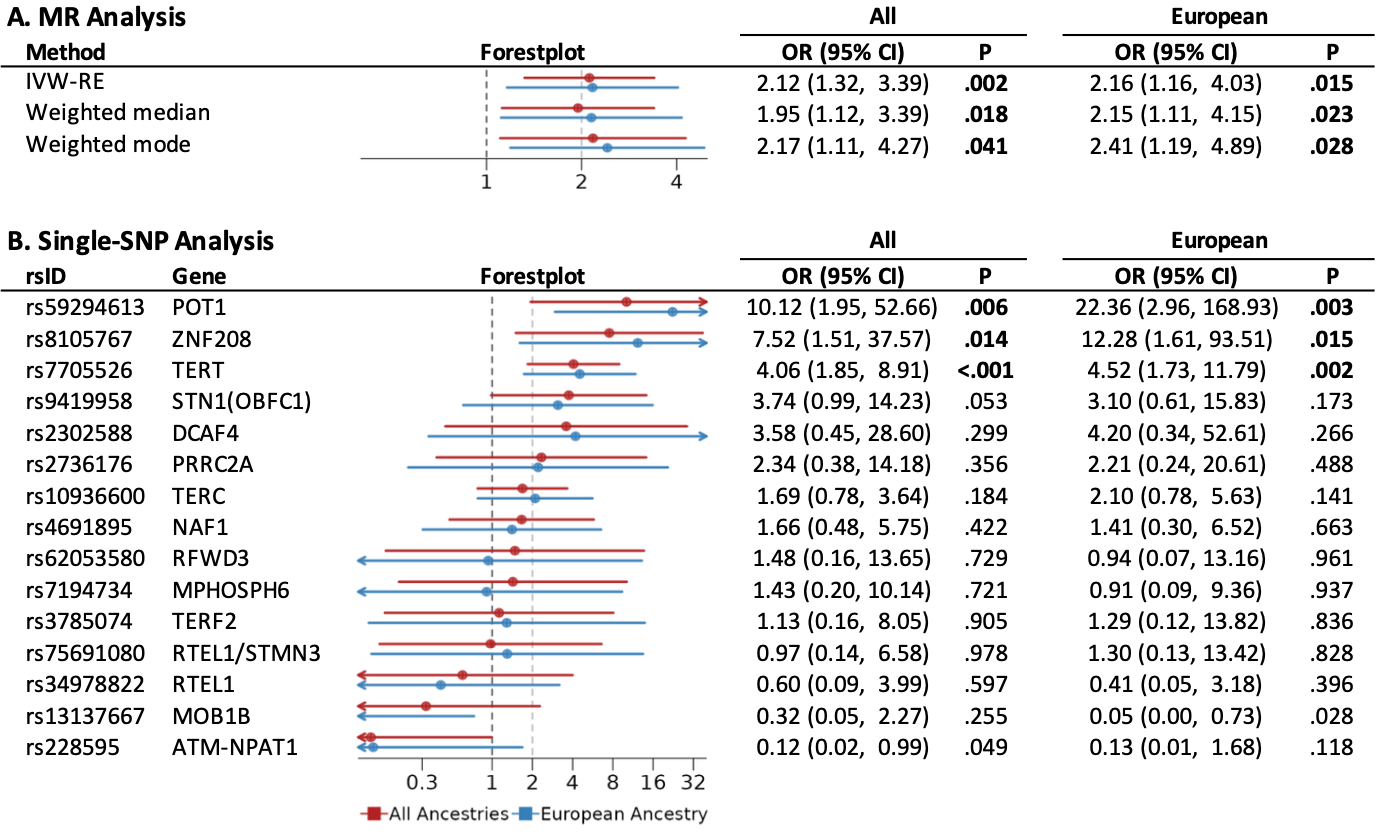


Figure S1 Forest plot of MR and single-SNP analysis of LTL effects on childhood glioma (all vs European ancestry), using 15-SNP genetic instrument. (A) Forest plot of overall MR estimates derived from inverse-variance weighted (random-effects, IVW-RE), weighted median, and weighted mode models. (B) Single-SNP analysis use Wald ratio test. Top three SNPs with highest OR are shown in bold. The dash black vertical line indicates the null value (ORSD = 1), and the grey dashed line marks an ORSD of 2. Red and blue denote results from all-ancestry and European ancestry analyses, respectively. Abbreviations: IVW-RE, inverse-variance weighted random-effects; MR, mendelian randomization; FDR, false discovery rate; ORSD, odds ratio per 1 standard deviation increase in the exposure.

## Figure S2. Forest plot of MR analysis of LTL effects on childhood glioma subtypes (all vs European ancestry)


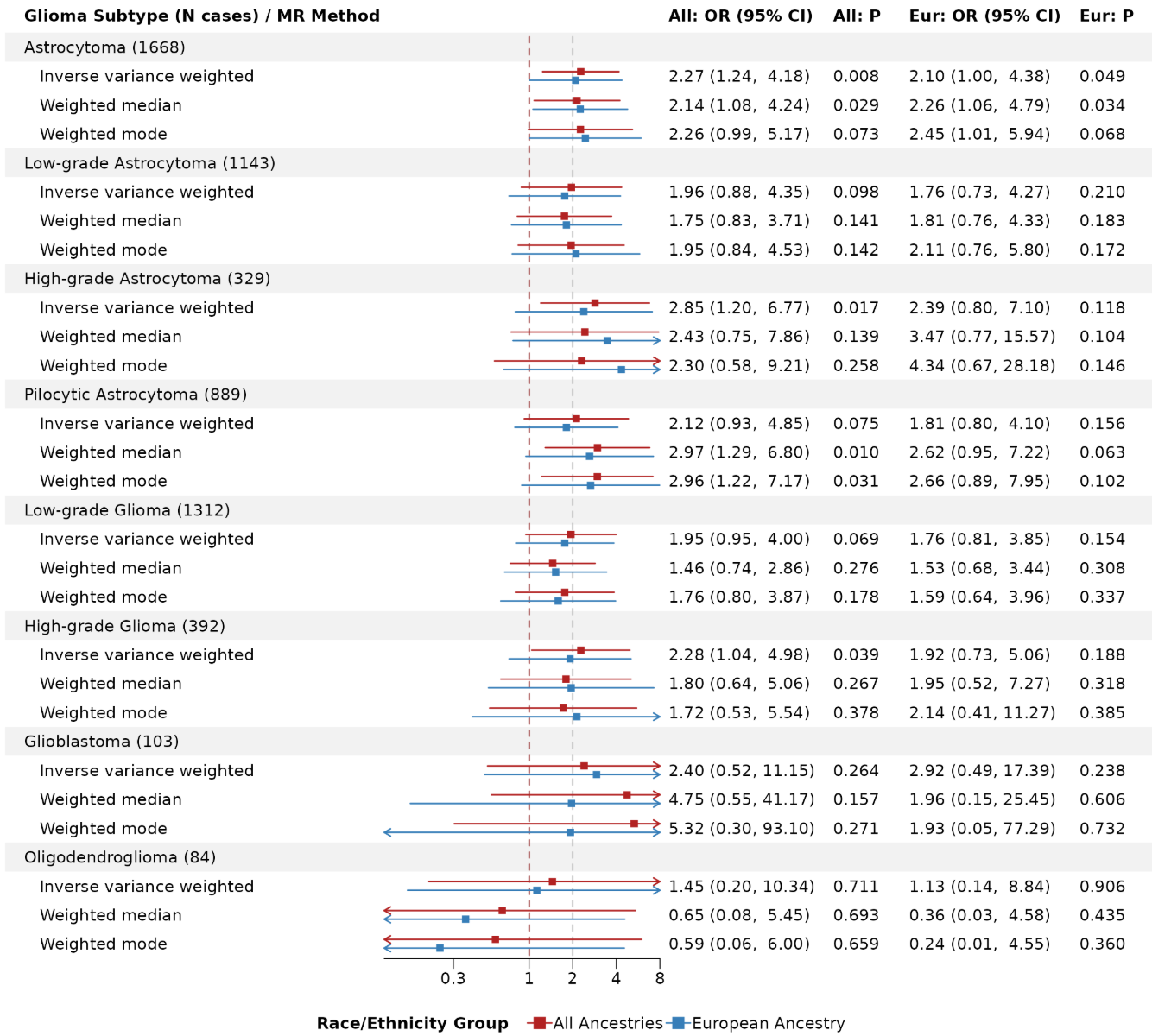


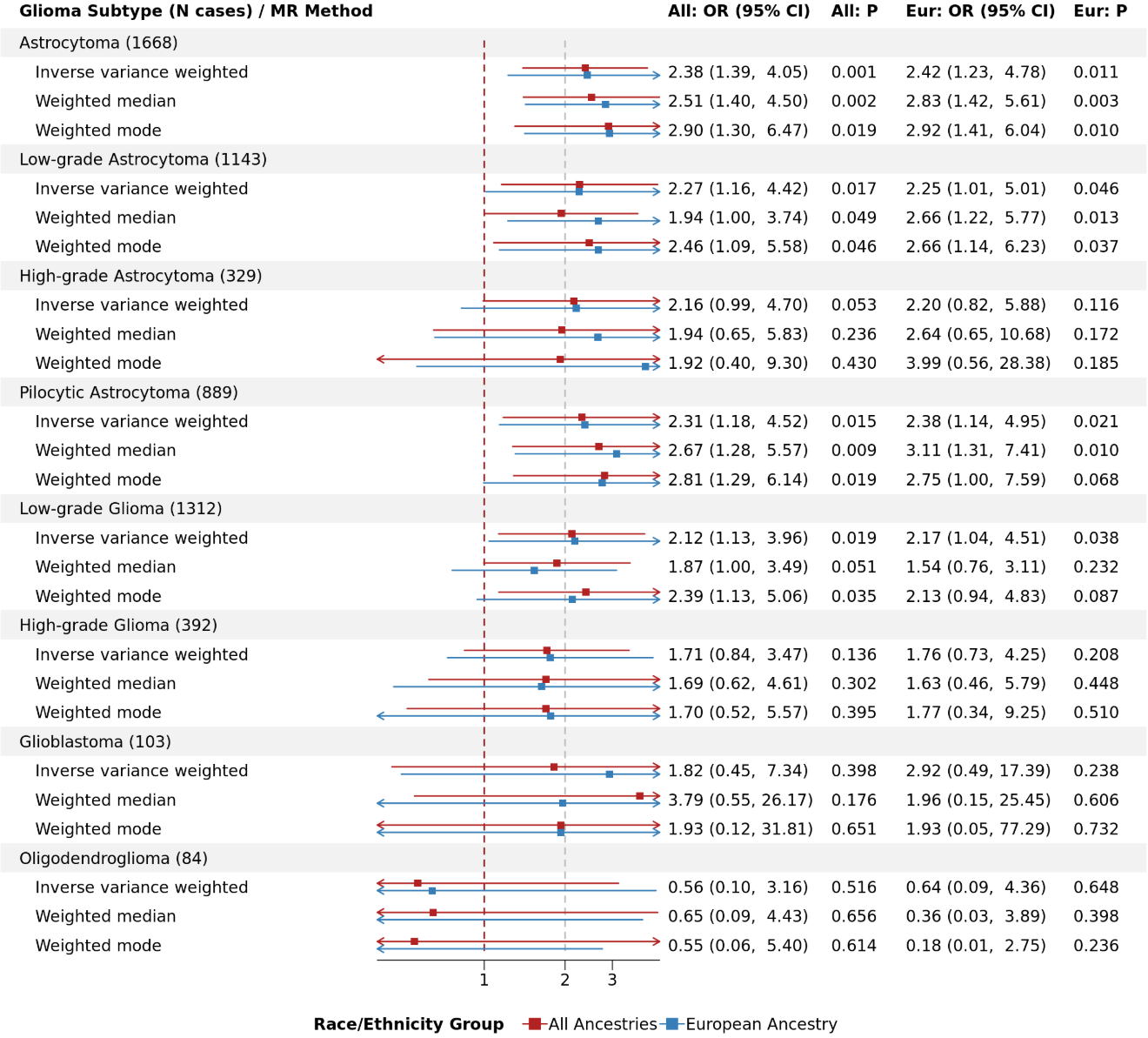


Figure S2. Forest plot of MR analysis of LTL effects on childhood glioma subtypes (all vs European ancestry). 15-SNP genetic instrument was used for analysis. MR estimates were derived using IVW-RE, weighted median, and weighted mode models. Results were reported as odds ratios (ORs) with 95% confidence intervals (CIs) per genetically predicted one standard deviation (SD) increase in directly measured telomere length. The dash black vertical line represents the null value (ORSD = 1), while the grey dashed line denotes an ORSD of 2. Red and blue denote results from all and European analyses, respectively. Abbreviations: IVW-RE, inverse-variance weighted random-effects; ORSD, odds ratio per 1 standard deviation increase in the exposure.

## Figure S3. Sensitivity MR analyses supporting main MR findings in Figure 2


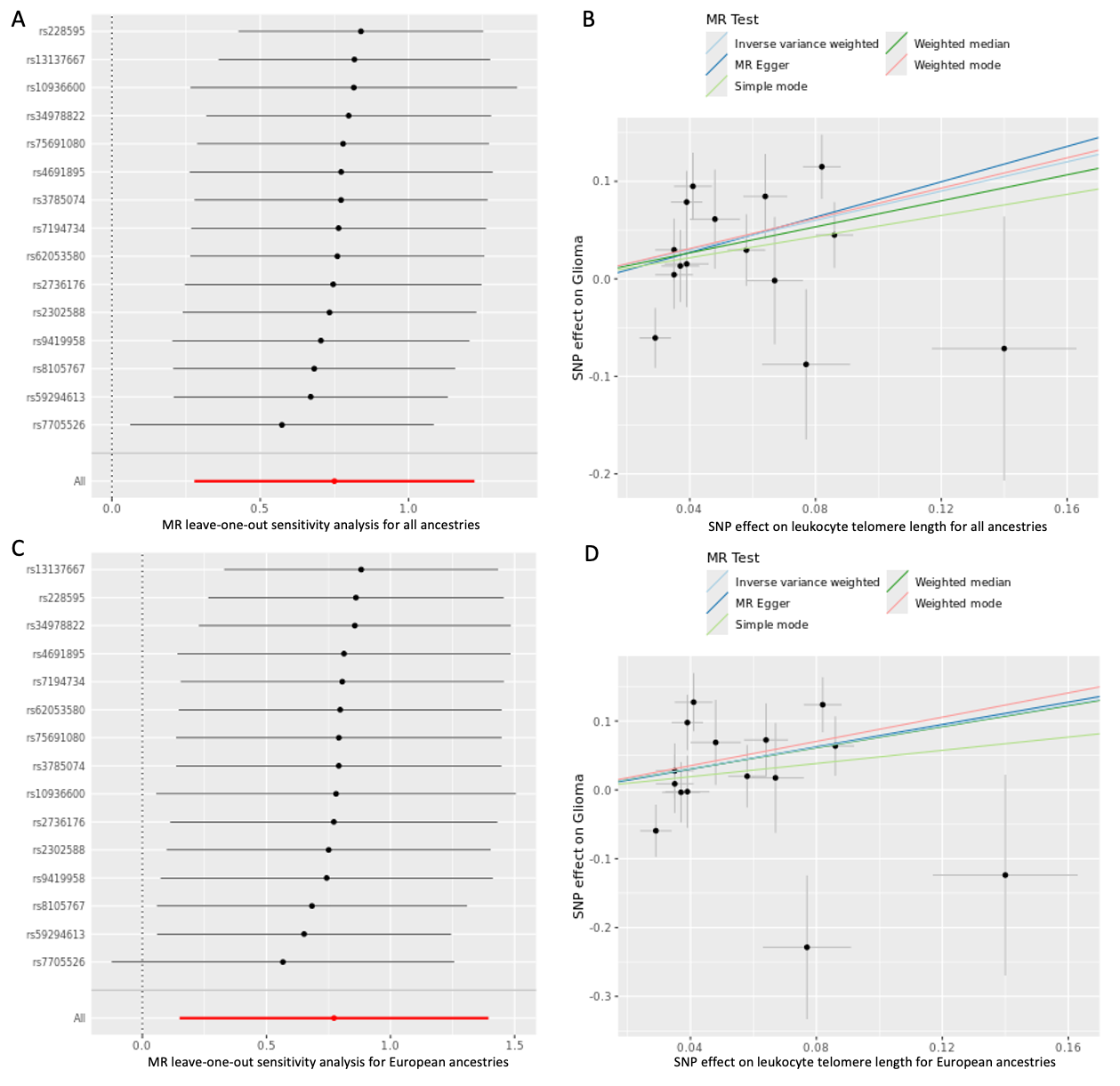


Figure S3 Sensitivity analyses corresponding to the main MR results presented in Figure 2 use 15-SNP genetic instrument.
(A) Leave-one-out (LOO) analysis for multi-ancestry group, showing the influence of individual SNPs on the overall causal estimate.
(B) Comparison of estimates across different MR models, including inverse-variance weighted (IVW), weighted median, weighted mode, simple mode, and MR Egger, for multi-ancestry group.
(C) Leave-one-out analysis for European ancestry group.
(D) Comparison of estimates across different MR models, including inverse-variance weighted (IVW), weighted median, weighted mode, and MR Egger, for European ancestry group.

Abbreviations: LOO, leave-one-out; MR, Mendelian randomization; IVW, inverse-variance weighted.

## Figure S4. MR-Clust results showing SNP clustering based on LTL and childhood glioma associations


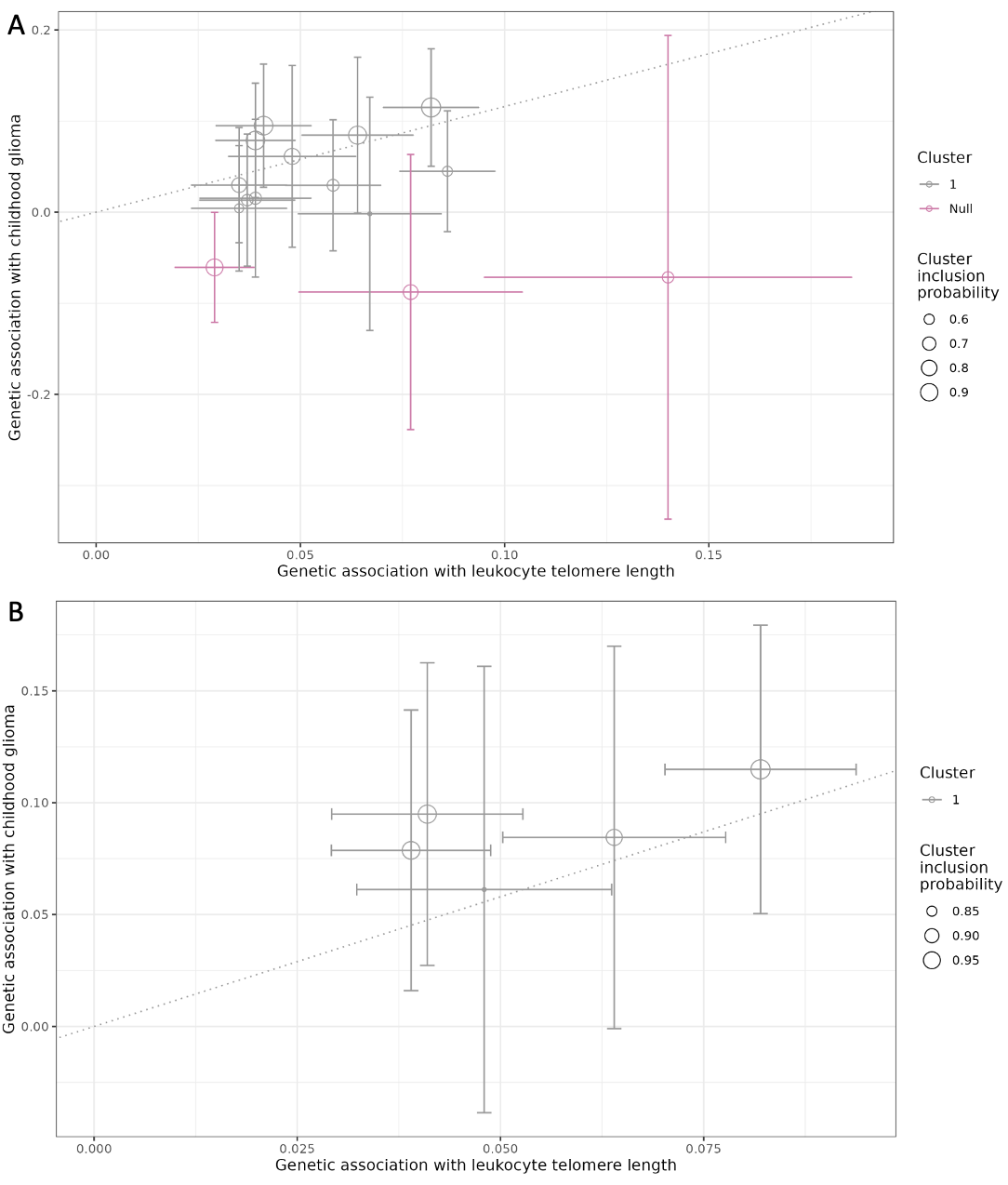


Figure S4 MR-Clust results showing 15 SNPs clustering based on LTL and childhood glioma associations. MR-Clust results illustrating the clustering of SNPs based on their genetic associations with LTL (x-axis) and childhood glioma risk (y-axis). (A) Results using all SNPs in the multi-ancestry analysis, with the best-fit clustering solution. SNPs are grouped into either a non-null cluster (grey) or a null cluster (pink). Error bars represent standard errors, and point size reflects the probability of inclusion in the assigned cluster. (B) Subset of SNPs from (A) with a cluster allocation probability greater than 0.8, highlighting those with the strongest support for inclusion in the non-null cluster. The dotted line in both panels represents the estimated causal effect direction.

## Figure S5 Density plots of LTL PRS by glioma status across four ancestry groups


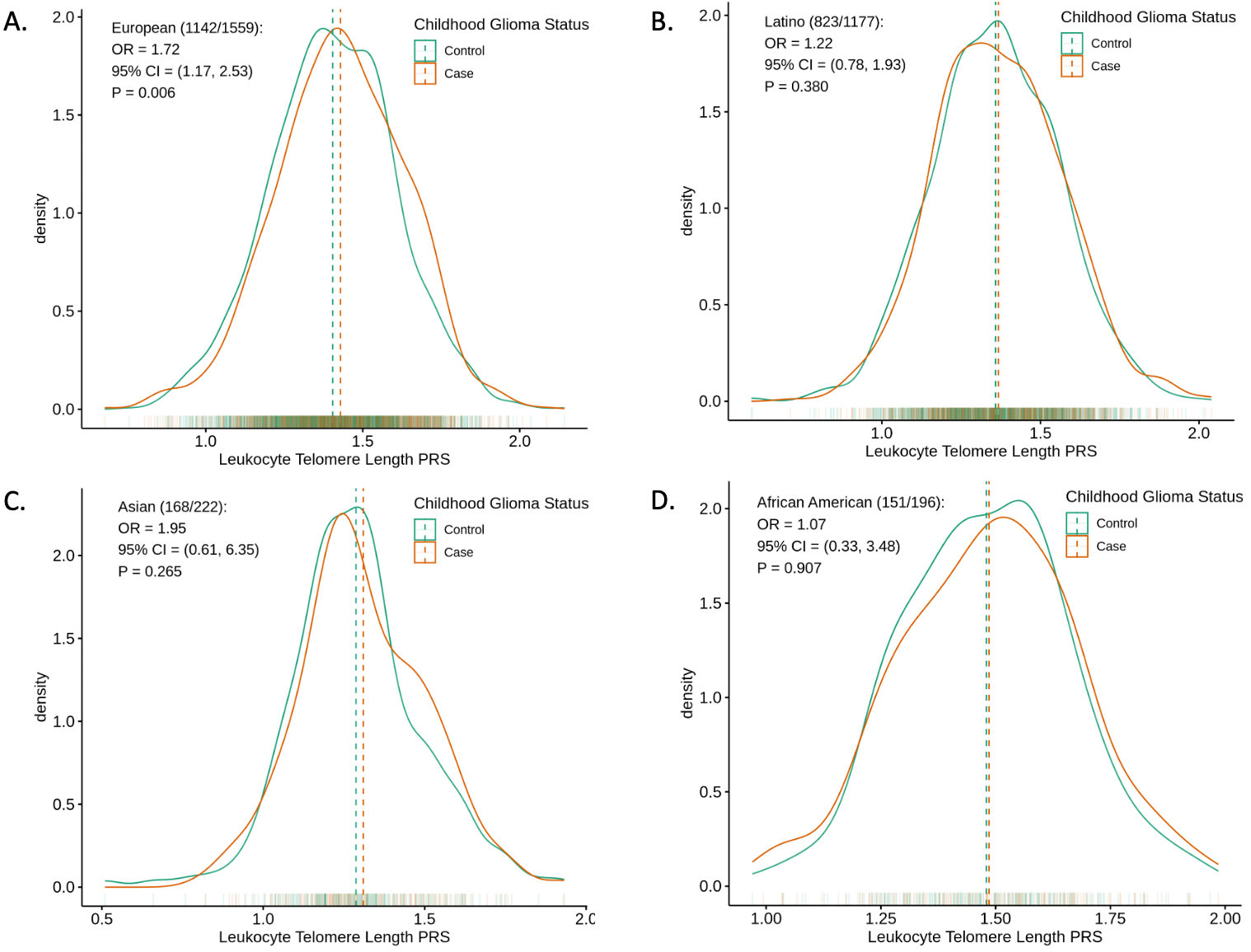


Figure S5 Density plots of LTL PRS by childhood glioma status across four ancestry groups: (A) European, (B) Latino, (C) Asian, and (D) African American. Orange and green lines represent childhood glioma cases and controls, respectively. Dashed vertical lines indicate the mean LTL PRS for each group. Odds ratios, 95% CIs, and *p*-values are shown for each ancestry group, based on logistic regression comparing LTL PRS between cases and controls and adjusting for sex and the first 10 genetic principal components.

Abbreviations: LTL PRS, leukocyte telomere length polygenic risk score.

## Figure S6. Sensitivity MR analyses supporting age-stratified MR findings in Figure 4A


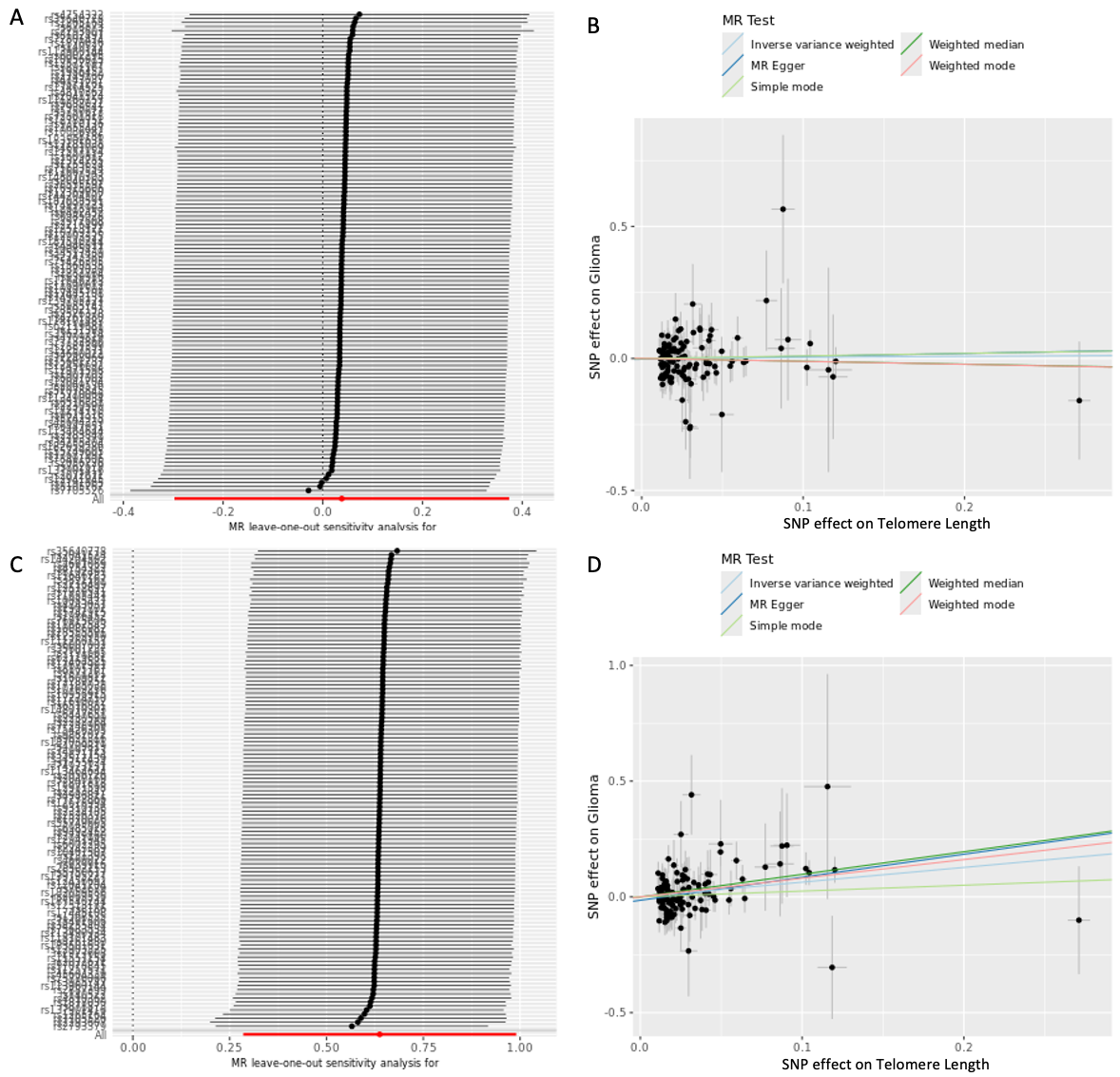


Figure S6 Sensitivity Analyses Supporting Age-stratified MR Findings in Figure 4A
(A) Leave-one-out (LOO) analysis for early-diagnosed group, showing the influence of individual SNPs on the overall causal estimate.
(B) Comparison of estimates across different MR models, including inverse-variance weighted (IVW), weighted median, weighted mode, and MR Egger, for early-diagnosed group.
(C) Leave-one-out analysis for later-diagnosed group.
(D) Comparison of estimates across different MR models, including inverse-variance weighted (IVW), weighted median, weighted mode, and MR Egger, for later-diagnosed group.

Abbreviations: LOO, leave-one-out; MR, Mendelian randomization; IVW, inverse-variance weighted.

| **Supplementary tables:**  \| Table S1. Demographic and phenotypic characteristics of the US cohort by ancestry group. \| \| \| \| \| --- \| --- \| --- \| --- \| \|  \| n (case/control) \| Age at diagnosis, median (Q25-Q75) \| Female, n (%) \| \| All \| 5495 (2310/3185) \| 6 (4-11) \| 2592 (47.2) \| \| Ancestry group \|  \|  \|  \| \| European \| 2701 (1142/1559) \| 7 (4-12) \| 1248 (46.2) \| \| Latino \| 2000 (823/1177) \| 6 (3-11) \| 973 (48.6) \| \| Asian \| 390 (168/222) \| 6 (4-12) \| 180 (46.2) \| \| African American \| 347 (151/196) \| 6 (4-11) \| 170 (49.0) \| \| Other \| 57 (26/31) \| 6 (4-12) \| 21 (36.8) \| \| Notes: The “Other ancestry” category includes 52 individuals of mixed ancestry, 2 American Indian participants, and 3 individuals with missing ancestry information. \| \| \| \|  Table S2. IVW heterogeneity test results | | | |
| --- | --- | --- | --- | --- | --- | --- | --- | --- | --- | --- | --- | --- | --- | --- | --- | --- | --- | --- | --- | --- | --- | --- | --- | --- | --- | --- | --- | --- | --- | --- | --- | --- | --- | --- | --- | --- | --- | --- | --- | --- | --- | --- | --- |
|  | Q | df | P |
| Multi-ancestry (all) | 23.62 | 14 | 0.05 |
| European ancestry | 27.03 | 14 | 0.02 |
| Age at diagnosis group: |  |  |  |
| Early diagnosed (<=6) | 119.94 | 117 | 0.41 |
| Later diagnosed (>6) | 107.75 | 118 | 0.74 |

Abbreviations: IVW, inverse-variance weighted; Q, Cochran’s Q; df, degree of freedom

| Table S3. Directional horizontal pleiotropy test | | | |
| --- | --- | --- | --- |
|  | Egger Intercept | SE | P |
| Multi-ancestry (all) | -0.018 | 0.035 | 0.63 |
| European ancestry | -0.006 | 0.048 | 0.90 |
| Age at diagnosis group: |  |  |  |
| Early diagnosed (<=6) | -0.003 | 0.009 | 0.74 |
| Later diagnosed (>6) | -0.015 | 0.009 | 0.12 |

Abbreviations: SE, standard error.

| Table S4. Logistic regression results for the association between LTL PRS and childhood glioma status, stratified by glioma subtypes. | | | |
| --- | --- | --- | --- |
|  | Childhood Glioma Status ~ LTL PRS + covariates | | |
|  | Leukocyte Telomere Length PRS | | |
| Glioma Subtype | N (case/control) | OR (95%CI) | P |
| Low-grade glioma | 1312 / 3185 | 1.29 (0.93, 1.79) | 0.128 |
| High-grade glioma | 392 / 3185 | 1.39 (0.81, 2.38) | 0.230 |
| Astrocytoma | 1668 / 3185 | 1.40 (1.03, 1.89) | 0.030 |
| Low-grade astrocytoma | 1143 / 3185 | 1.28 (0.90, 1.80) | 0.164 |
| high-grade astrocytoma | 329 / 3185 | 1.48 (0.83, 2.65) | 0.184 |
| Pilocytic astrocytoma | 889 / 3185 | 1.19 (0.82, 1.74) | 0.366 |
| Glioblastoma | 103 / 3185 | 1.66 (0.60, 4.56) | 0.326 |
| Oligodendroglioma | 84 / 3185 | 2.39 (0.79, 7.27) | 0.123 |
| Abbreviations: LTL PRS, leukocyte telomere length polygenic risk score; OR, odds ratio; CIs, confidence intervals; AIC, Akaike Information Criterion; PCs, principal components. Notes: Odds ratios (ORs) per one-unit increase in LTL PRS were estimated using logistic regression models adjusted for sex and the first 10 genetic PCs. ORs are presented with 95% confidence intervals (CIs) and corresponding P values. | | | |
